# Supplementary material for: A Likelihood Approach for Real-Time Calibration of Stochastic Compartmental Epidemic Models
Source: PLoS Comput Biol. 2017 Jan 17;13(1):e1005257. doi: 10.1371/journal.pcbi.1005257 (PMC5240920; doi:10.1371/journal.pcbi.1005257)
Supplement: S1 File — (TAR.GZ) [file pcbi.1005257.s014.tar.gz › HSPH_Online-SI-Revision/PF08/n1-severe/n1-severe_PF_table4.pdf]

1	R0	{1.51081, 13.7078}	{1.62771, 14.1564}	{{1.23374, 8.14982}, {2.16158, 19.7928}}	{20.6528, 24.5031}	inf	60.	42.0272	{4.07864, 87.5221}	52.0132	0.385556	newly	28.	17.59	{4., 32.}	47.3929	0.376177	Reff	0.861162	0.717143	{0.400856, 0.977481}	23.1864	ar	656.	744.67	{534., 977.}	23.1326
2	R0	{1.73182, 11.3873}	{1.66542, 14.1738}	{{1.24689, 7.41497}, {2.24029, 20.}}	{19.2602, 38.8436}	inf	73.	58.3699	{17.9713, 128.75}	51.6294	0.340508	newly	43.	24.6	{8., 45.}	49.1163	0.372794	Reff	0.82781	0.702889	{0.405405, 0.921356}	21.5695	ar	653.	767.31	{525., 984.}	25.3063
3	R0	{1.50449, 10.3474}	{1.68119, 14.7704}	{{1.23525, 8.22878}, {2.3669, 20.}}	{23.3381, 51.3657}	inf	61.	55.0589	{11.233, 106.889}	52.1239	0.322032	newly	34.	23.1	{6., 43.}	45.3529	0.331631	Reff	0.825965	0.734985	{0.389458, 1.05045}	23.417	ar	651.	752.52	{497., 997.}	26.9002
4	R0	{1.63493, 9.1955}	{1.87647, 14.2197}	{{1.25959, 7.43049}, {2.76838, 19.876}}	{26.1172, 63.2578}	inf	51.	71.7169	{25.7196, 136.959}	70.199	0.237206	newly	40.	33.21	{13., 56.}	38.375	0.213742	Reff	0.734084	0.847341	{0.513501, 1.17086}	29.472	ar	663.	824.96	{572., 1044.}	29.8824
5	R0	{1.53822, 8.05553}	{1.67848, 13.5419}	{{1.13239, 7.48339}, {2.2277, 19.1171}}	{23.5797, 75.1879}	inf	25.	46.7649	{3.821, 112.742}	133.656	0.456613	newly	19.	20.34	{2., 41.}	68.1053	0.394596	Reff	0.772186	0.713826	{0.422011, 0.943499}	24.1103	ar	621.	615.64	{294., 852.}	27.3591
6	R0	{1.69374, 17.0793}	{1.58154, 14.6751}	{{1.19173, 7.40074}, {2.10237, 20.}}	{20.4762, 21.4636}	inf	95.	50.8386	{4.81832, 115.684}	56.9276	0.516753	newly	35.	20.76	{4., 40.}	51.4286	0.422053	Reff	0.98745	0.743402	{0.447511, 0.963659}	25.2281	ar	662.	705.39	{478., 951.}	24.3066
7	R0	{1.42179, 11.8031}	{1.68337, 14.5593}	{{1.22284, 7.72832}, {2.36592, 19.9053}}	{25.8365, 37.7559}	inf	48.	43.0419	{10.7673, 77.4803}	53.1571	0.33991	newly	29.	17.48	{4., 30.}	48.4828	0.377608	Reff	0.796202	0.654005	{0.327584, 0.901221}	24.9046	ar	517.	767.38	{520., 1005.}	50.3172
8	R0	{1.46683, 6.95875}	{2.05976, 15.1752}	{{1.28801, 9.71573}, {2.78092, 20.}}	{44.3338, 120.937}	inf	30.	84.5577	{33.6413, 156.737}	186.751	0.40124	newly	30.	35.24	{16., 55.}	45.2667	0.173753	Reff	0.545661	0.777563	{0.473128, 1.11883}	48.9425	ar	653.	887.22	{611., 1046.}	40.1562
9	R0	{1.34525, 11.505}	{1.62244, 13.9394}	{{1.20826, 7.69843}, {2.12133, 19.8874}}	{25.7276, 38.8062}	inf	49.	39.947	{4.05212, 85.844}	55.4903	0.398895	newly	38.	16.74	{4., 33.}	58.3158	0.493231	Reff	0.881139	0.68198	{0.432984, 0.917425}	25.803	ar	532.	743.4	{506., 959.}	42.0902
10	R0	{1.77958, 14.372}	{1.73579, 14.7678}	{{1.26114, 8.47036}, {2.40577, 20.}}	{22.1268, 25.6961}	inf	58.	48.9772	{11.8584, 90.9949}	51.4962	0.322684	newly	28.	19.75	{6., 36.}	43.75	0.31008	Reff	0.726069	0.643765	{0.309389, 0.886086}	26.0192	ar	671.	789.87	{545., 1012.}	25.8912
11	R0	{1.60334, 19.9548}	{1.57419, 13.9719}	{{1.17862, 7.11618}, {2.10226, 20.}}	{19.2821, 30.0322}	inf	78.	29.9744	{0.603124, 63.7998}	64.1084	0.737773	newly	24.	12.41	{0., 29.}	56.7917	0.644063	Reff	0.928334	0.669715	{0.396331, 0.851834}	28.2065	ar	617.	711.51	{496., 945.}	26.1572
12	R0	{1.68623, 7.58585}	{1.87633, 13.8865}	{{1.27357, 7.55154}, {2.73737, 19.1609}}	{25.3695, 88.8357}	inf	21.	54.5895	{9.5169, 96.9959}	181.342	0.437438	newly	21.	23.1	{7., 42.}	54.8571	0.262501	Reff	0.696413	0.645333	{0.326845, 0.950669}	29.9528	ar	640.	685.44	{389., 908.}	27.175
13	R0	{1.82689, 18.0022}	{1.66371, 15.4258}	{{1.19387, 7.76681}, {2.31252, 20.}}	{21.3067, 20.4774}	inf	97.	50.8863	{11.3939, 99.0594}	52.4751	0.438415	newly	31.	20.1	{5., 35.}	44.0645	0.33717	Reff	0.811139	0.685074	{0.386817, 0.962351}	24.189	ar	673.	756.43	{501., 991.}	24.8366
14	R0	{1.75911, 9.16883}	{1.87158, 14.1541}	{{1.31707, 8.39294}, {2.56757, 19.0269}}	{22.6226, 60.1907}	inf	49.	71.2523	{13.3114, 143.15}	86.0768	0.333155	newly	35.	31.13	{7., 62.}	55.1143	0.309692	Reff	0.809191	0.681874	{0.350507, 0.930751}	25.8539	ar	661.	704.62	{437., 898.}	22.1331
15	R0	{1.46168, 11.3859}	{1.66368, 14.3571}	{{1.19047, 8.2453}, {2.20082, 19.8864}}	{22.7961, 39.9001}	inf	42.	41.0912	{3.98628, 80.683}	60.6606	0.39376	newly	28.	16.7	{2., 33.}	50.7857	0.437638	Reff	0.69576	0.665018	{0.36778, 0.883221}	21.8836	ar	556.	762.14	{525., 992.}	40.1475
16	R0	{1.53577, 11.7581}	{1.74389, 13.4443}	{{1.15964, 6.62607}, {2.52006, 19.1994}}	{24.9162, 34.9235}	inf	70.	55.7966	{3.57163, 132.587}	63.3568	0.509994	newly	40.	24.56	{2., 52.}	58.55	0.505392	Reff	0.7771	0.723349	{0.37283, 0.967048}	24.9954	ar	625.	648.78	{362., 884.}	25.1808
17	R0	{1.70698, 9.72924}	{1.84416, 13.5246}	{{1.13227, 7.48339}, {2.46976, 18.4749}}	{23.6551, 48.5814}	inf	45.	61.09	{5.44261, 130.268}	84.9115	0.39953	newly	22.	26.4	{3., 54.}	65.8182	0.337103	Reff	0.626462	0.677362	{0.350507, 0.959454}	31.4715	ar	675.	669.55	{290., 887.}	25.7052
18	R0	{1.5973, 14.931}	{1.61136, 12.949}	{{1.11228, 5.71232}, {2.27582, 18.9813}}	{24.3948, 29.5838}	inf	48.	44.6481	{2.69113, 95.6764}	65.2212	0.481921	newly	24.	20.52	{2., 43.}	56.0833	0.408664	Reff	1.13089	0.797489	{0.431869, 1.08208}	35.6581	ar	632.	573.2	{259., 866.}	30.5127
19	R0	{1.87468, 17.004}	{1.7168, 15.0617}	{{1.20721, 7.72916}, {2.37411, 20.}}	{22.0393, 21.5748}	inf	76.	37.003	{6.57405, 70.9577}	59.8496	0.505857	newly	35.	14.04	{3., 28.}	61.2571	0.540117	Reff	0.783616	0.600377	{0.307364, 0.835381}	26.5798	ar	692.	779.14	{543., 1013.}	23.8728
20	R0	{1.84715, 12.531}	{1.69935, 14.7511}	{{1.26409, 8.95867}, {2.3731, 20.}}	{20.873, 33.7217}	inf	47.	38.4701	{5.16846, 72.1047}	53.4115	0.389473	newly	27.	15.63	{2., 30.}	49.	0.427338	Reff	0.718541	0.622766	{0.311091, 0.859415}	23.3795	ar	661.	772.31	{538., 996.}	25.3843
21	R0	{1.75938, 13.5959}	{1.6824, 13.1038}	{{1.14345, 5.72868}, {2.31174, 19.1171}}	{21.6223, 29.1373}	inf	73.	49.2589	{1.98736, 103.678}	57.5084	0.540018	newly	35.	22.73	{2., 47.}	54.5429	0.47389	Reff	0.932471	0.723327	{0.363322, 0.943958}	28.1154	ar	620.	613.65	{314., 855.}	26.65
22	R0	{1.63456, 10.7469}	{1.74889, 15.2332}	{{1.24128, 9.13438}, {2.37573, 20.}}	{20.4824, 50.8908}	inf	44.	46.8654	{10.1827, 87.2506}	59.3412	0.32267	newly	29.	18.34	{5., 34.}	46.8966	0.379215	Reff	0.626036	0.617365	{0.339444, 0.864961}	26.1886	ar	654.	791.22	{527., 984.}	29.2997
23	R0	{1.62702, 19.1392}	{1.79721, 14.3153}	{{1.2772, 7.00618}, {2.38773, 20.}}	{23.4964, 26.5742}	inf	90.	41.1434	{11.4104, 89.98}	59.8868	0.479078	newly	31.	18.15	{8., 33.}	48.3548	0.333452	Reff	0.810256	0.825165	{0.512881, 1.18845}	22.9398	ar	600.	773.67	{529., 1005.}	34.105
24	R0	{1.70466, 12.1202}	{1.7892, 14.5334}	{{1.24839, 7.69038}, {2.62773, 20.}}	{23.2179, 35.9368}	inf	45.	31.4435	{7.05816, 72.9547}	54.6853	0.36915	newly	23.	14.18	{4., 31.}	51.913	0.369172	Reff	0.758574	0.757972	{0.468803, 1.01578}	23.8342	ar	613.	769.14	{538., 1002.}	31.4845
25	R0	{1.61735, 11.3169}	{1.68131, 15.4812}	{{1.2716, 7.91254}, {2.27352, 20.}}	{19.3753, 45.1842}	inf	43.	36.1359	{4.34834, 80.8726}	56.2752	0.390929	newly	31.	14.01	{2., 26.}	56.6129	0.543168	Reff	0.608124	0.620793	{0.367898, 0.83641}	24.7665	ar	681.	769.2	{557., 965.}	20.884
26	R0	{1.56977, 12.3035}	{1.74643, 12.9873}	{{1.14345, 5.72868}, {2.31231, 19.0531}}	{24.2558, 32.6825}	inf	36.	44.061	{1.61748, 118.177}	93.7813	0.536638	newly	18.	18.87	{2., 43.}	74.1667	0.442742	Reff	0.846106	0.665126	{0.382133, 0.941978}	29.0424	ar	594.	639.66	{359., 871.}	30.3367
27	R0	{1.46317, 5.8298}	{1.9154, 15.1653}	{{1.30384, 8.39098}, {2.75968, 20.}}	{35.2321, 161.075}	inf	14.	61.955	{19.2122, 119.878}	344.788	0.558012	newly	14.	26.46	{9., 48.}	103.143	0.282472	Reff	0.626237	0.755083	{0.430718, 1.11374}	35.0034	ar	586.	840.51	{567., 1030.}	46.4898
28	R0	{1.70002, 14.9806}	{1.69571, 13.6588}	{{1.15689, 7.48339}, {2.27287, 19.2972}}	{21.7194, 24.8704}	inf	101.	62.2917	{3.74857, 143.315}	56.1379	0.510402	newly	48.	27.83	{3., 62.}	55.2708	0.487892	Reff	0.85511	0.791859	{0.433202, 1.0551}	23.028	ar	652.	617.67	{314., 862.}	26.5506
29	R0	{1.52729, 16.1014}	{1.65069, 14.4984}	{{1.20248, 7.70935}, {2.39664, 20.}}	{21.9958, 21.4103}	inf	46.	33.1341	{5.81291, 62.3215}	49.9951	0.392339	newly	17.	13.71	{2., 23.}	42.7647	0.341295	Reff	0.781972	0.649749	{0.302332, 0.887813}	24.0092	ar	592.	747.09	{521., 1002.}	31.603
30	R0	{1.72795, 13.5635}	{1.77471, 14.3629}	{{1.28324, 8.29975}, {2.36663, 19.6963}}	{20.0868, 26.1413}	inf	61.	42.5769	{3.48679, 80.5124}	56.9528	0.470361	newly	28.	16.46	{2., 33.}	53.2143	0.44947	Reff	0.661805	0.616598	{0.33392, 0.876776}	25.2527	ar	668.	807.93	{578., 1015.}	27.1841
31	R0	{1.51591, 18.1544}	{1.64969, 13.8926}	{{1.23376, 6.9864}, {2.14391, 19.9732}}	{20.6292, 27.2191}	inf	51.	41.8495	{2.44414, 86.3987}	59.3861	0.483784	newly	16.	17.03	{3., 35.}	62.0625	0.395247	Reff	0.950476	0.704898	{0.437207, 0.975473}	27.7324	ar	541.	749.59	{515., 982.}	40.7634
32	R0	{1.4282, 10.3534}	{1.67805, 13.5144}	{{1.20292, 6.40075}, {2.30604, 19.7952}}	{24.5953, 47.4801}	inf	33.	48.7362	{5.83467, 95.7034}	88.8166	0.37553	newly	26.	21.1	{5., 37.}	44.3846	0.307526	Reff	1.03687	0.69673	{0.41016, 0.94542}	33.3384	ar	521.	754.06	{489., 966.}	48.0422
33	R0	{1.56693, 11.3972}	{1.87304, 13.5779}	{{1.21278, 6.78013}, {2.49423, 19.3966}}	{30.0253, 37.2837}	inf	84.	65.4869	{7.72364, 132.587}	56.8084	0.468836	newly	50.	27.58	{4., 53.}	56.	0.508093	Reff	0.634607	0.630628	{0.35021, 0.879573}	25.8782	ar	695.	697.71	{362., 897.}	22.9971
34	R0	{1.50675, 19.8823}	{1.65525, 14.5562}	{{1.18832, 8.14454}, {2.19461, 19.9186}}	{21.7884, 26.9102}	inf	63.	30.6634	{3.70594, 68.2724}	60.3481	0.517643	newly	29.	12.89	{2., 26.}	59.9655	0.546056	Reff	0.797071	0.673782	{0.406943, 0.923518}	23.5346	ar	587.	756.22	{522., 985.}	33.8637
35	R0	{1.72889, 7.76068}	{1.90968, 14.5683}	{{1.30044, 8.42022}, {2.46976, 19.255}}	{23.1575, 91.6061}	inf	43.	71.9391	{11.3391, 130.268}	93.2459	0.348116	newly	34.	29.77	{8., 54.}	42.0294	0.276931	Reff	0.7002	0.657279	{0.377161, 0.929894}	26.7416	ar	650.	715.69	{441., 881.}	25.2846
36	R0	{1.62644, 4.12303}	{1.93809, 13.2152}	{{1.27231, 7.55154}, {2.59602, 18.4504}}	{29.0173, 220.596}	inf	8.	50.0923	{9.66278, 103.84}	532.29	0.691594	newly	9.	22.01	{6., 41.}	160.333	0.387035	Reff	0.57088	0.589651	{0.29678, 0.950096}	36.0346	ar	652.	719.85	{408., 903.}	26.5322
37	R0	{1.70878, 15.8072}	{1.63928, 15.0555}	{{1.24307, 7.77119}, {2.06364, 20.}}	{18.4672, 23.8768}	inf	45.	29.9738	{4.092, 56.4827}	53.3581	0.437683	newly	13.	11.83	{2., 23.}	50.6923	0.345949	Reff	0.772369	0.622471	{0.389758, 0.831826}	23.5442	ar	674.	754.64	{558., 953.}	22.184
38	R0	{1.58967, 6.80568}	{2.02493, 14.4815}	{{1.32901, 7.44114}, {2.74007, 20.}}	{34.5058, 115.72}	inf	25.	62.1037	{16.8542, 116.529}	160.733	0.368574	newly	22.	27.26	{11., 48.}	53.3636	0.203888	Reff	0.661303	0.786339	{0.486054, 1.14573}	32.5599	ar	610.	850.88	{586., 1018.}	42.8852
39	R0	{1.344, 8.19498}	{1.63765, 14.3084}	{{1.20283, 7.07695}, {2.27376, 20.}}	{27.2782, 83.4751}	inf	37.	41.1493	{5.26054, 84.52}	61.6978	0.355442	newly	28.	16.77	{4., 30.}	48.1071	0.399829	Reff	0.756672	0.685961	{0.363034, 0.963491}	23.4235	ar	551.	732.37	{480., 986.}	38.176
40	R0	{1.61509, 7.90825}	{1.79827, 12.9907}	{{1.12977, 6.74711}, {2.46533, 17.5057}}	{27.4567, 69.624}	inf	33.	52.5628	{3.23339, 109.743}	107.683	0.433293	newly	23.	23.38	{3., 44.}	62.6957	0.370185	Reff	0.814005	0.675739	{0.372595, 1.06243}	31.2586	ar	581.	647.48	{243., 879.}	37.2702
41	R0	{1.60017, 5.90605}	{2.11068, 15.1656}	{{1.34337, 8.33192}, {2.78296, 20.}}	{37.6631, 159.057}	inf	23.	76.9518	{19.3435, 172.171}	242.933	0.457538	newly	19.	31.23	{11., 61.}	82.4737	0.242148	Reff	0.611265	0.686324	{0.40263, 0.98812}	33.0841	ar	661.	901.01	{608., 1047.}	40.2859
42	R0	{1.64436, 15.1337}	{1.62363, 12.8333}	{{1.13227, 5.72868}, {2.1968, 18.4749}}	{21.8321, 28.316}	inf	68.	38.3665	{1.61748, 89.1508}	63.6493	0.670585	newly	31.	16.51	{1., 38.}	62.0323	0.621322	Reff	0.902754	0.730342	{0.448023, 1.0327}	28.6623	ar	640.	577.91	{256., 845.}	28.8141
43	R0	{1.48917, 11.917}	{1.55073, 13.832}	{{1.18762, 6.98078}, {2.01624, 20.}}	{17.4867, 37.2984}	inf	22.	27.296	{0.90071, 56.581}	86.8532	0.497181	newly	12.	10.93	{1., 24.}	62.4167	0.474597	Reff	0.814576	0.662178	{0.415488, 0.847227}	20.6428	ar	551.	707.18	{481., 906.}	33.6044
44	R0	{1.65577, 7.89317}	{1.78799, 13.4394}	{{1.15964, 6.62607}, {2.52651, 19.1171}}	{25.3187, 78.9139}	inf	38.	55.0783	{3.57477, 132.689}	101.232	0.458239	newly	25.	24.	{2., 55.}	63.84	0.39017	Reff	0.819606	0.700679	{0.333599, 0.948216}	26.7201	ar	576.	646.61	{314., 894.}	35.158
45	R0	{1.6909, 12.3275}	{1.87763, 15.1144}	{{1.24903, 8.96987}, {2.47968, 20.}}	{23.6553, 34.4502}	inf	39.	48.8921	{20.0073, 82.121}	55.914	0.211974	newly	24.	21.14	{9., 34.}	41.5833	0.213716	Reff	0.749069	0.774678	{0.50103, 1.06559}	25.8062	ar	675.	821.18	{559., 1008.}	29.1526
46	R0	{1.62351, 16.0698}	{1.62361, 14.2262}	{{1.19636, 8.18447}, {2.39551, 20.}}	{22.0203, 23.4952}	inf	55.	40.8514	{9.06786, 78.7118}	50.394	0.343835	newly	26.	18.5	{5., 32.}	45.4615	0.320324	Reff	1.091	0.695557	{0.359675, 0.937362}	36.2459	ar	591.	729.54	{517., 995.}	29.5127
47	R0	{1.52372, 4.38599}	{2.19955, 14.5307}	{{1.50998, 8.35312}, {3., 19.7316}}	{47.1813, 231.931}	inf	12.	93.8791	{26.0339, 157.148}	682.326	0.801586	newly	15.	40.12	{18., 64.}	170.667	0.392316	Reff	0.594251	0.703722	{0.41812, 1.00036}	35.2677	ar	618.	905.46	{682., 1067.}	48.6149
48	R0	{1.44306, 11.788}	{1.78023, 14.2257}	{{1.2391, 6.41738}, {2.35922, 19.4366}}	{29.3862, 37.8798}	inf	18.	46.1658	{13.1705, 91.0818}	170.617	0.384923	newly	11.	20.82	{7., 37.}	107.091	0.304294	Reff	0.793683	0.792701	{0.508592, 1.07914}	23.0708	ar	534.	791.92	{529., 997.}	51.0861
49	R0	{1.63624, 11.3893}	{1.87758, 13.8293}	{{1.21937, 8.26611}, {2.52651, 19.2972}}	{27.6217, 35.2556}	inf	48.	51.9065	{7.33857, 109.725}	70.2949	0.406406	newly	31.	21.4	{5., 48.}	55.4194	0.428715	Reff	0.713401	0.605468	{0.329032, 0.860511}	27.7034	ar	643.	696.3	{386., 899.}	26.4821
50	R0	{1.46113, 11.1322}	{1.66019, 13.949}	{{1.20062, 7.25364}, {2.38843, 19.7054}}	{24.4066, 42.4314}	inf	30.	52.1233	{8.19379, 98.8138}	108.013	0.364783	newly	18.	21.87	{6., 36.}	59.0556	0.269376	Reff	0.936584	0.725343	{0.387269, 1.03045}	26.7827	ar	501.	732.85	{458., 996.}	49.2954
